# Analysis of Factors Associated with Early Childhood Caries in Jinan City: A Cross-Sectional Study

**DOI:** 10.3290/j.ohpd.c_2830

**Published:** 2026-10-30

**Authors:** Yuke Shang, Chengmei Zhang, Jia Chang, Yang Yu, Dandan Liu, Lixia Zhang

**Affiliations:** a Yuke Shang Master’s Student in Dentistry, Department of Pediatric Dentistry, Binzhou Medical University, Yantai, Shandong Province, China. Jinan Stomatological Hospital, Jinan, Shandong Province, China; Central Laboratory, Jinan Key Medical and Health Laboratory of Oral Diseases and Tissue Regeneration, Jinan Key Laboratory of Oral Diseases and Tissue Regeneration, Shandong Provincial Key Medical and Health Laboratory of Oral Diseases and Tissue Regeneration, Jinan, Shandong Province, China. Conceived and designed the study, collected and analyzed the data, drafted the initial manuscript, and finalized the revision.; b Chengmei Zhang Physician, Department of Pediatric Dentistry, Jinan Stomatological Hospital, Jinan, Shandong Province, China; Central Laboratory, Jinan Key Medical and Health Laboratory of Oral Diseases and Tissue Regeneration, Jinan Key Laboratory of Oral Diseases and Tissue Regeneration, Shandong Provincial Key Medical and Health Laboratory of Oral Diseases and Tissue Regeneration, Jinan, Shandong Province, China. Participated in data organization, statistical analysis, and figure/table preparation.; c Jia Chang Primary Nurse, Department of Pediatric Dentistry, Jinan Stomatological Hospital, Jinan, Shandong Province, China. Assisted in experimental operations, data validation, and manuscript proofreading.; d Yang Yu Attending Physician, Department of Pediatric Dentistry, Jinan Stomatological Hospital, Jinan, Shandong Province, China; Central Laboratory, Jinan Key Medical and Health Laboratory of Oral Diseases and Tissue Regeneration, Jinan Key Laboratory of Oral Diseases and Tissue Regeneration, Shandong Provincial Key Medical and Health Laboratory of Oral Diseases and Tissue Regeneration, Jinan, Shandong Province, China. Provided technical support, contributed to discussion of results, and checked references.; e Dandan Liu Attending Physician, Department of Pediatric Dentistry, Jinan Stomatological Hospital, Jinan, Shandong Province, China; Central Laboratory, Jinan Key Medical and Health Laboratory of Oral Diseases and Tissue Regeneration, Jinan Key Laboratory of Oral Diseases and Tissue Regeneration, Shandong Provincial Key Medical and Health Laboratory of Oral Diseases and Tissue Regeneration, Jinan, Shandong Province, China. Assisted in project management and critically revised the manuscript.; f Lixia Zhang Chief Physician, Department of Pediatric Dentistry, Jinan Stomatological Hospital, Jinan, Shandong Province, China; Central Laboratory, Jinan Key Medical and Health Laboratory of Oral Diseases and Tissue Regeneration, Jinan Key Laboratory of Oral Diseases and Tissue Regeneration, Shandong Provincial Key Medical and Health Laboratory of Oral Diseases and Tissue Regeneration, Jinan, Shandong Province, China. Guided research direction, provided funding support, reviewed and approved the manuscript.

**Keywords:** caries risk factors, caries susceptibility, correlation analysis, early childhood caries.

## Abstract

**Purpose:**

Early childhood caries (ECC) remains a global public health challenge characterized by high prevalence and low treatment rates. This study investigated factors associated with ECC among 3- to 5-year-old children in Jinan through oral examinations, questionnaires, and caries activity testing, aiming to support early prevention.

**Materials and Methods:**

A total of 733 children aged 3-5 in Jinan were enrolled. Sociodemographic information, feeding patterns, and oral health behaviors were collected via questionnaires completed by legal guardians. The number of decayed, missing, and filled surfaces (dmfs) was recorded. Oral plaque was collected for Cariostat caries activity testing. Statistical analyses were conducted to identify factors associated with ECC and the relationship between ECC and caries activity.

**Results:**

Among 733 participants, the prevalence of ECC was 76.8%. Factors statistically significantly associated with caries severity included age, gender, primary caregiver’s educational attainment, frequency of sweet-snack consumption, and the habit of nighttime milk feeding or falling asleep with a nipple (all p < 0.05). The proportion of high-risk children (by Cariostat) progressively increased across the caries-free, low-caries, and high-caries groups (p < 0.001).

**Conclusion:**

Low educational attainment of the primary caregiver, inappropriate feeding practices, frequent sugary food intake, and poor oral hygiene habits are risk factors for ECC. Oral health education and promotion of proper oral hygiene habits are therefore needed. Furthermore, the acid‑producing metabolic activity of oral plaque microorganisms increased significantly with caries progression, suggesting that Cariostat test values may serve as a potential indicator of cumulative caries severity in preschool children.

Early childhood caries (ECC), defined by the American Academy of Pediatric Dentistry as the presence of one or more decayed, missing (due to caries), or filled primary tooth surfaces (dmfs) in children under 71 months of age, is the most prevalent chronic disease in early childhood globally.^26^ It is a non-communicable disease closely associated with behavioral habits, lifestyle, and microbial dysbiosis. Rather than being caused by a specific pathogenic bacterium, ECC results from a dysbiosis of the dental biofilm—an ecological imbalance induced by frequent exposure to fermentable carbohydrates that favors the proliferation of aciduric and acidogenic species over health-associated commensals.^11,15^

Affecting approximately 48% of preschool-aged children worldwide,^30^ ECC poses a substantial public health burden. In China, the Fourth National Oral Health Epidemiological Survey reported ECC prevalence rates of 50.8%, 63.6%, and 71.9% among 3-, 4-, and 5-year-old children, respectively. Notably, the prevalence in 5-year-olds has risen by 5.9 percentage points over the past decade, while the treatment rate remains critically low at 4.0%.^6^ The consequences of ECC extend beyond primary dentition. Affected children have a 5- to 6-fold higher risk of caries in permanent teeth and potential disruptions in dental development.^23^ Clinical management often requires physical restraint or general anesthesia due to young age and behavioral challenges, causing psychological distress and imposing financial strain on families. Moreover, recurrence rates after conventional treatment range from 22% to 79% when etiological factors persist.^1,28,38^ As a multifactorial condition driven by dietary patterns, oral microbial ecology, socioeconomic status, and hygiene behaviors, ECC is most strongly associated with modifiable risks—particularly frequent sugar exposure and inadequate toothbrushing practices. Because these risks are largely preventable, evidence-based, etiology-targeted prevention is urgently needed.

Within China, ECC prevalence shows marked spatiotemporal heterogeneity, with rising trends over time and pronounced regional disparities. Notably, higher prevalence is often observed in economically advanced coastal regions, revealing a disconnect between socioeconomic development and pediatric oral health outcomes.^17,37,39^ Shandong Province—consistently among China’s top three economies—and its capital Jinan (which surpassed a GDP of ¥1 trillion in 2020) exemplify this disparity. Despite robust economic indicators, children’s oral health remains under-prioritized. Data from the Fourth National Oral Health Epidemiological Survey confirm this concern: Shandong’s average ECC prevalence among 3- to 5-year-olds reached 64.6% (exceeding the national average of 61.8%),^6^ and a 2018 Jinan-specific survey reported 59.4% prevalence in the same age group, indicating a persistent public health crisis.^27^ In response, Shandong Province has launched initiatives including the Implementation Plan for Healthy Oral Action in Shandong Province (2019–2025) and the Medium- and Long-Term Plan for Chronic Disease Prevention and Control in Shandong Province (2017–2025). Jinan City has begun implementing pit-and-fissure sealants and early caries interventions for eligible preschoolers. However, the practical effectiveness of these policies remains unquantified at the local level, and contemporary risk factor profiles specific to Jinan’s evolving sociodemographic context are lacking. Rigorous, localized evidence is therefore essential to guide policy refinement and resource allocation.

Accordingly, this study aims to: (1) evaluate the preliminary impact of current oral health policies on ECC prevalence in Jinan City; (2) update the epidemiological profile of ECC and identify predominant modifiable risk factors among preschool children in the region; and (3) generate evidence-based recommendations to optimize targeted prevention strategies and inform future policy development in Jinan and similar settings.

## MATERIALS AND METHODS

### Research Subjects and Ethical Approval

A total of 733 children aged 3 to 5 years were recruited from six randomly selected kindergartens in Jinan City, Shandong Province. The inclusion criteria were as follows: (1) aged 3 to 5 years old; (2) in good general health without systemic diseases; and (3) provision of parents’ or guardian’s written informed consent. The exclusion criteria included: (1) antibiotic use or fluoride treatment within the past two weeks; and (2) inability to cooperate during oral examination or sample collection.

The study was approved by the Ethics Committee of Jinan Stomatological Hospital (Approval No.: JNSKQYY-2023-011) and was conducted in accordance with the Declaration of Helsinki. Written informed consent was obtained from the legal guardians of all participating children.

### Oral Health Questionnaire

A structured questionnaire was designed based on the version used for children aged 3 to 5 years in the Manual of the Fourth National Oral Health Epidemiological Survey in China. The questionnaires were uniformly distributed by kindergarten teachers to the guardians. Those who agreed to participate in this study signed an informed consent form and completed the questionnaire. Univariate analysis was applied to questionnaire responses from the three groups to identify factors associated with ECC.

### Oral Examination

All oral examinations were conducted by two experienced pediatric dentists. Inter-examiner reliability was confirmed with a Kappa coefficient of 0.84. Examinations were performed with children in a semi-recumbent position under natural light, using a disposable plain mouth mirror and a No. 5 explorer. dmfs scores were recorded according to the diagnostic criteria outlined in the WHO publication Oral Health Surveys: Basic Methods (ed 5, 2013).

### Caries Severity Classification

Caries severity in this study was defined according to the cumulative caries experience measured by dmfs scores, rather than lesion depth or clinical stage. Based on dmfs values, children were categorized into three groups: caries-free (dmfs = 0), low-caries (dmfs = 1–4), and high-caries (dmfs > 4).^31^ This classification was used for subsequent statistical analyses to investigate associations between caries severity and potential risk factors.

### Caries Activity Testing 

Oral plaque samples were collected between 8:00 and 10:30 a.m. Children were instructed not to brush teeth on the morning of sampling and to abstain from eating or drinking for at least 90 minutes prior to collection. Caries activity testing was performed using the Cariostat caries susceptibility test kit (Hebei Gangda Kaiwei Biotechnology; Hebei, China). A dedicated swab from the Cariostat kit was used to wipe the buccal side of maxillary molars and the labial cervical area of mandibular anterior teeth 3-5 times to collect plaque. The plaque was inoculated into the culture medium and placed in a constant-temperature incubator at 37°C ±1°C for 48 h within a 4-h window post-collection. Under natural light, the color of the culture medium was compared with the standard color chart provided by the manufacturer to read the Cariostat value, as shown in Fig 1. Then, caries susceptibility was classified based on the Cariostat value (Table 1).

**Fig 1:**
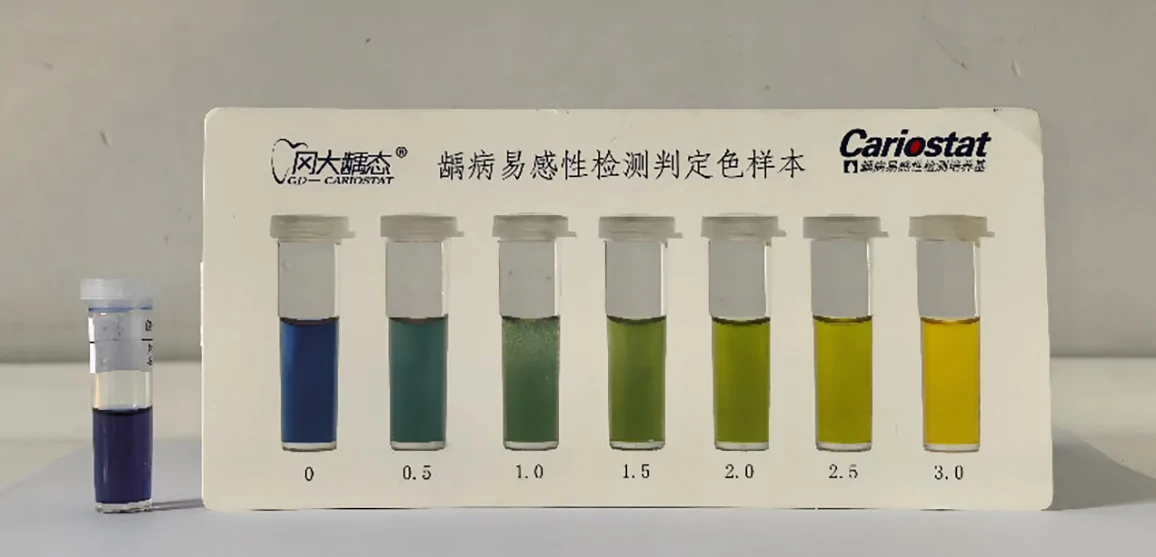
Reading of Cariostat values.

**Table 1:** Distribution of Cariostat values

Value	Color	pH range	Susceptibility
0	Blue	>6.0	Safe zone
0.5	Dark green	5.9-5.7	Safe zone
1.0	Light green	5.6-5.2	Caution zone
1.5	Yellow-green	5.1-4.9	Risk zone
2.0	Light yellow-green	4.8-4.6	Risk zone
2.5	Light yellow	4.5-4.3	Risk zone
3.0	Yellow	<4.2	Risk zone


### Statistical Analysis

Data analysis was conducted using SPSS version 22.0 (IBM; Armonk, NY, USA). All statistical tests were two-tailed, with a significance level set at α = 0.05. The Kolmogorov-Smirnov and Shapiro-Wilk tests were applied to assess data normality. Normally distributed continuous variables were presented as mean ± standard deviation, while categorical variables were described using frequencies and percentages. The chi-squared test was used to compare categorical variables across groups. For ranked data among the three groups, the Kruskal-wallis test was employed. Furthermore, ordinal logistic regression was performed to identify factors associated with ECC caries severity.

## RESULTS

### Prevalence of Dental Caries and Distribution of Caries Severity 

A total of 1011 questionnaires were distributed, and 851 were returned. After excluding participants who did not meet the eligibility criteria, 733 children aged 3-5 years were included in the final analysis. According to the dmfs-based caries severity classification described above, 170 children (23.2%) were classified as caries-free, while 563 children had ECC, yielding an overall caries prevalence of 76.8%. Among children with ECC, 153 (20.9% of all participants) were categorized into the low-caries group, and 410 (55.9% of all participants) were classified as the high-caries group. Subsequent analyses were performed to compare sociodemographic characteristics, feeding behaviors, oral hygiene practices, and caries activity among the three caries severity groups.

### Comparison of Sociodemographic Characteristics among the Three Groups

As shown in Table 2, statistically significant differences in age, sex, and primary caregiver educational attainment were observed among the three caries-status groups. The proportion of 5-year-old children increased from 32.4% in the caries-free group to 50.2% in the high-caries group (p < 0.001), indicating that older children were more frequently represented in the high-caries group. The proportion of boys was also higher in the high-caries group than in the caries-free and low-caries groups (55.4%, 44.1%, and 46.4%, respectively; p = 0.022). In addition, the proportion of primary caregivers with a bachelor’s degree or higher was lower in the high-caries group (39.3%) than in the caries-free (54.7%) and low-caries (52.9%) groups (p < 0.001). No statistically significant difference in primary caregiver type was observed among the three groups (p = 0.905).

**Table 2:** Comparison of sociodemographic information among the three groups of children

Variable	Caries-free group (n = 170)	Low caries group (n = 153)	High caries group (n = 410)	X^2^ value	p-value
Age in years*				16.005	<0.001
3	15 (8.8)	16 (10.5)	25 (6.1)		
4	100 (58.8)	74 (48.4)	179 (43.7)		
5	55 (32.4)	63 (41.2)	206 (50.2)		
**Gender**				7.638	0.022
Boy	75 (44.1)	71 (46.4)	227 (55.4)		
Girl	95 (55.9)	82 (53.6)	183 (44.6)		
**Primary caregiver**				3.422	0.905
Father	12 (7.1)	9 (5.9)	22 (5.4)		
Mother	106 (62.4)	101 (66.0)	249 (60.7)		
Grandparents (paternal/maternal)	26 (15.3)	23 (15.0)	79 (19.3)		
Father and mother	25 (14.7)	19 (12.4)	56 (13.7)		
Mother and (paternal/maternal) grandparents	1 (0.6)	1 (0.7)	4 (1.0)		
Educational attainment of primary caregiver*				16.519	<0.001
Primary school and below	4 (2.4)	5 (3.3)	31 (7.6)		
Junior high school	15 (8.8)	11 (7.2)	44 (10.7)		
High school/ secondary technical school	26 (15.3)	21 (13.7)	68 (16.6)		
Junior college	32 (18.8)	35 (22.9)	106 (25.9)		
Bachelor’s degree and above	93 (54.7)	81 (52.9)	161 (39.3)		
* Kruskal-Wallis test for multiple independent samples.					

### Comparison of Feeding Habits among the Three Groups

The comparison of feeding habits among the three groups is presented in Table 3. No statistically significant difference was observed in feeding methods within the first 6 months after birth among the groups (p = 0.065). However, statistically significant differences were found in the frequency of eating sweet snacks (p = 0.001), frequency of drinking sweet beverages (p = 0.013), bedtime consumption of milk, beverages, or sweet snacks (p = 0.009), and the habit of night-time milk feeding or sleeping with a nipple in the mouth (p < 0.001). Compared with the caries-free and low-caries groups, children in the high-caries group were more likely to consume sweet snacks frequently, consume milk, beverages, or sweet snacks before bedtime, and have night-time milk feeding or sleeping with a nipple. In the questionnaire, “milk” referred to milk feeding as reported by caregivers; the type of milk and whether it contained added sugar or other carbohydrates were not further distinguished.

**Table 3:** Comparison of feeding habits among the three groups of children

Variable	Caries-free group (n = 170)	Low caries group (n = 153)	High caries group (n = 410)	X^2^ value	p-value
Feeding method within 6 months after birth*				5.476	0.065
Exclusive breastfeeding	66 (38.8)	62 (40.5)	188 (45.9)		
Mainly breastfeeding	42 (24.7)	43 (28.1)	109 (26.6)		
Half breastfeeding and half artificial feeding	44 (25.9)	34 (22.2)	93 (22.7)		
Mainly artificial feeding	9 (5.3)	8 (5.2)	9 (2.2)		
Exclusive artificial feeding	9 (5.3)	6 (3.9)	11 (2.7)		
Frequency of eating sweet snacks*				13.331	0.001
≥ 2 times a day	8 (4.7)	10 (6.5)	35 (8.5)		
1 time a day	26 (15.3)	20 (13.1)	78 (19.0)		
2 – 6 times a week	65 (38.2)	54 (35.3)	168 (41.0)		
1 time a week	32 (18.8)	40 (26.1)	75 (18.3)		
1 – 3 times a month	33 (19.4)	29 (19.0)	51 (12.4)		
Never	6 (3.5)	0 (0.0)	3 (0.7)		
Frequency of drinking sweet beverages*				8.682	0.013
≥ 2 times a day	2 (1.2)	1 (0.7)	8 (2.0)		
1 time a day	9 (5.3)	1 (0.7)	12 (2.9)		
2 – 6 times a week	17 (10.0)	16 (10.5)	63 (15.4)		
1 time a week	26 (15.3)	35 (22.9)	98 (23.9)		
1 – 3 times a month	81 (47.6)	74 (48.4)	168 (41.0)		
Never	35 (20.6)	26 (17.0)	61 (14.9)		
Habit of drinking milk or other beverages, or eating sweet snacks before going to bed at night*				9.370	0.009
1 time a day	25 (14.7)	16 (10.5)	69 (16.8)		
2 – 6 times a week	28 (16.5)	22 (14.4)	83 (20.2)		
1 time a week	24 (14.1)	16 (10.5)	42 (10.2)		
1 – 3 times a month	29 (17.1)	38 (24.8)	97 (23.7)		
Never	64 (37.6)	61 (39.9)	119 (29.0)		
Habit of drinking milk at night or sleeping with a nipple in the mouth*				77.395	<0.001
Often	3 (1.8)	7 (4.6)	108 (26.3)		
Occasionally	135 (79.4)	138 (90.2)	279 (68.0)		
Never	32 (18.8)	8 (5.2)	23 (5.6)		
* Kruskal-Wallis test for multiple independent samples.					

### Comparison of Oral Hygiene Habits and Dental Visit Situations among the Three Groups

The comparison of oral hygiene habits and dental visit situations among the three groups is presented in Table 4. No statistically significant differences were observed in age at brushing initiation (p = 0.132) or use of fluoride toothpaste (p = 0.121) among the groups. However, statistically significant differences were found in toothbrushing frequency (p = 0.029), parental assistance with dental floss use (p = 0.022), dental visit experience (p = 0.007), and the reason for the child’s most recent dental visit (p < 0.001). Compared with the caries-free and low-caries groups, children in the high-caries group were less likely to brush their teeth twice or more per day, less likely to receive regular parental assistance with dental floss use, more likely to have visited a dentist, and more likely to seek dental care for treatment-related reasons rather than preventive care.

**Table 4:** Comparison of oral hygiene habits and dental visit situations among the three groups of children

Variable	Caries-free group (n = 170)	Low caries group (n = 153)	High caries group (n = 410)	X^2^ Value	p-value
Age when starting to brush teeth*				4.052	0.132
6 – 12 months	24 (14.1)	18 (11.8)	31 (7.6)		
1 – 2 years	45 (26.5)	44 (28.8)	108 (26.3)		
2 – 3 years	51 (30.0)	47 (30.7)	135 (32.9)		
3 – 4 years	38 (22.4)	29 (19.0)	98 (23.9)		
4 – 5 years	7 (4.1)	9 (5.9)	27 (6.6)		
5 years and above	3 (1.8)	6 (3.9)	8 (2.0)		
Never brushes teeth	2 (1.2)	0 (0.0)	3 (0.7)		
Frequency of daily toothbrushing *				7.092	0.029
2 times and above	43 (25.3)	43 (28.1)	70 (17.1)		
1 time	90 (52.9)	77 (50.3)	236 (57.6)		
Not every day	37 (21.8)	33 (21.6)	104 (25.4)		
**Use of fluoride toothpaste**				10.088	0.121
Yes	93 (54.7)	97 (63.4)	202 (49.3)		
No	44 (25.9)	31 (20.3)	110 (26.8)		
Don’t know	33 (19.4)	25 (16.3)	97 (23.7)		
Don’t use toothpaste	0 (0.0)	0 (0.0)	1 (0.2)		
Frequency of parents helping children use dental floss*				7.654	0.022
Don’t use	117 (68.8)	100 (65.4)	310 (75.6)		
Occasionally use	49 (28.8)	45 (29.4)	94 (22.9)		
Use every week	4 (2.4)	3 (2.0)	4 (1.0)		
Use every day	0 (0.0)	5 (3.3)	2 (0.5)		
**Has the child visited a dentist?**				9.909	0.007
Yes	82 (48.2)	85 (55.6)	255 (62.2)		
Never	88 (51.8)	68 (44.4)	155 (37.8)		
**Reason for the child’s most recent dental visit**				39.078	<0.001
Consultation/seeking advice	10 (5.9)	22 (14.4)	59 (14.4)		
Preventive care	51 (30.0)	40 (26.1)	92 (22.4)		
Emergency treatment for oral pain or discomfort, etc.	16 (9.4)	13 (8.5)	90 (22.0)		
Non-emergency treatment	9 (5.3)	13 (8.5)	32 (7.8)		
Don’t know/can’t remember	84 (49.4)	65 (42.5)	137 (33.4)		
* Kruskal-Wallis test for multiple independent samples.					

### Multivariate Analysis of Risk Factors for Early Childhood Caries

The results of the ordinal logistic regression analysis are presented in Table 5. Age, male sex, primary caregiver educational attainment, frequency of sweet-snack consumption, and the habit of nighttime milk feeding or falling asleep with a nipple in the mouth were independently associated with caries severity. Among these factors, frequent nighttime milk feeding or sleeping with a nipple showed the strongest association with increased caries severity (β = 3.236, p < 0.001), followed by daily sweet snack consumption (β = 1.631, p = 0.040). In contrast, higher educational attainment of the primary caregiver was associated with lower caries severity. These findings suggest that both unfavorable feeding behaviors and family socioeconomic characteristics contribute to the progression of ECC.

**Table 5:** Results of ordered multinomial logistic regression analysis of factors influencing the occurrence of low and high dental caries in children

Variable	Estimated value β	p- value	OR	95% confidence interval for β	
				Lower bound	Upper bound
Age	0.421	0.001	1.52	0.173	0.669
Gender (ref: female)					
Male	0.433	0.007	1.54	0.117	0.748
Educational attainment of the main caregiver (ref: Bachelor’s degree and above)					
Primary school and below	-1.387	0.001	0.25	-2.227	-0.547
Junior high school	-0.763	0.085	0.47	-1.632	0.106
High school / secondary technical school	-0.892	0.050	0.41	-1.786	0.002
Junior college	-0.763	0.118	0.47	-1.720	0.195
Frequency of sweet snacks (ref: never)					
≥ 2 times a day	1.541	0.070	4.67	-0.125	3.207
1 time a day	1.631	0.040	5.11	0.071	3.191
2 – 6 times a week	1.408	0.069	4.10	-0.110	2.926
1 time a week	1.181	0.129	3.26	-0.344	2.706
1 – 3 times a month	0.783	0.312	2.19	-0.734	2.301
Habit of drinking milk at night or falling asleep with the nipple in the mouth (ref: never)					
Often	3.236	<0.001	25.43	2.370	4.101
Occasionally	0.835	0.002	2.30	0.305	1.365


### Results of Oral Plaque Caries Activity Testing

The distribution of Cariostat-based caries activity differed statistically significantly among the three groups (χ² = 96.73, p < 0.001) (Table 6). The proportion of children classified as high-risk increased progressively from the caries-free group to the low-caries and high-caries groups. Consistently, Cariostat values showed a statistically significantly increasing trend with increasing caries severity (p < 0.001; Table 7). These findings suggest that Cariostat-based caries activity was associated with ECC severity.

**Table 6:** Distribution of cariostat caries risk among the three groups of children

Cariostat value	All	Caries-free group (n=67)	Low-caries group (n=81)	High-caries group (n=154)	X^2^ value	p-value
Safe zone (0.0-0.5)	16 (5.3)	9 (13.4)	5 (6.2)	2 (1.3)		
Caution zone (1.0)	70 (23.2)	38 (56.7)	25 (30.9)	7 (4.5)	96.738	<0.001
Risk zone (1.5-3.0)	216 (71.5)	20 (29.9)	51 (63.0)	145 (94.2)		
Kruskal-Wallis test for multiple independent samples.						

**Table 7:** Comparison of Cariostat values among the three groups of children

Group	Cariostat value	F value	p-value
Caries-free group (n=67)	1.13±0.41		
Low-caries group (n=81)	1.41±0.45*	96.165	<0.001
High-caries group (n=154)	1.96±0.45*#		
* p < 0.05 compared with the caries-free group; # p < 0.05 compared with the low-caries group.			

### Availability of Data and Materials

Data related to the study results are accessible through the corresponding author on request.

## DISCUSSION

As a cross-sectional investigation, the study provides an updated overview of ECC among preschool children in Jinan. The prevalence of ECC was 76.8%, exceeding the national average (62.1%) reported in the Fourth National Oral Health Epidemiological Survey in China. Participants were predominantly recruited from older urban districts of Jinan: long-established areas with mature infrastructure, healthcare services, and public facilities. Compared with newly developed districts, these older districts have a relatively backward economy and lower oral health awareness. Although this sampling strategy may have introduced some selection bias, the findings highlight that substantial gaps in ECC prevention remain even in areas with relatively good access to healthcare resources. Improving children’s oral health therefore requires not only access to dental services but also stronger community-based prevention programs and oral health education.

Among children aged 3–5 years, caries severity increases with age, consistent with previous studies.^2,13,39^ As primary teeth erupt and remain exposed to fermentable carbohydrates for longer periods, cumulative demineralization increases the likelihood of caries development.^7,14^ These findings support the importance of establishing preventive measures early in life, including regular dental examinations, fluoride application, and timely management of early lesions.

The association between sex and ECC remains controversial. Earlier studies suggested that girls may be at higher risk of caries because of earlier tooth eruption and longer exposure of tooth surfaces to cariogenic challenges. However, more recent epidemiological studies have reported inconsistent findings, with some showing no statistically significant sex differences in ECC prevalence.^16,20,36^ In the present study, boys were more likely to present with more severe caries than girls. A similar finding was reported by Shaffer et al^29^ in a Northern Appalachian population, although the authors emphasized that the mechanisms underlying this difference remain unclear. Given the cross-sectional nature of the present study, no conclusions can be drawn regarding the biological, behavioral, or sociocultural pathways responsible for the observed association. Further studies are required to clarify whether sex is an independent determinant of ECC or a surrogate marker for other social, behavioral, environmental, or biological factors.

Primary caregiver education was identified as an important social determinant of ECC. Consistent with previous studies,^9,25,35^ caregivers with higher educational attainment generally demonstrate better oral health awareness and greater engagement in preventive practices. These findings suggest that oral health promotion programs should prioritize families with lower educational levels and employ communication strategies that are simple, practical, and easily understood.

Dietary behaviors were strongly associated with caries severity. Frequent consumption of sweet snacks may promote the growth and metabolic activity of cariogenic bacteria, increasing acid production and enamel demineralization.^7,14,28,34^ Therefore, reducing children’s intake of sugary foods and beverages should remain a key target of oral health promotion and health policy.

Nighttime feeding behaviors, including consumption of milk, beverages or sweet snacks before sleep, nighttime milk feeding, and falling asleep with a nipple in the mouth, were associated with greater caries severity. However, this finding should not be interpreted as evidence that milk itself increases ECC risk. Current evidence indicates that milk and dairy products are generally non-cariogenic and may even contribute to oral health through buffering and remineralization effects.^32^ Instead, the observed association may reflect prolonged nocturnal exposure to fermentable carbohydrates, reduced salivary flow during sleep, and inadequate oral hygiene following feeding. Because the present questionnaire did not distinguish among different types of milk or beverages, future studies should further investigate the role of feeding composition and sugar content in nighttime feeding practices.

No association was observed between ECC and feeding patterns during the first six months of life. This finding suggests that feeding practices may exert their greatest influence after tooth eruption, when dental surfaces become susceptible to cariogenic challenges. Although evidence regarding optimal feeding methods after tooth eruption remains inconclusive, long-term breastfeeding ≥2 years and high-frequency breastfeeding have been identified as potential risk factors in previous studies.^8,12,24^

Proper oral hygiene remains fundamental for ECC prevention. Our results suggest that the consistency and quality of oral hygiene practices are crucial.^21^ Children often lack the manual dexterity required for effective plaque removal, making parental supervision and assistance essential. In addition, parental support for interdental cleaning may help reduce proximal plaque accumulation and caries development in primary dentition.^10^ The finding that most children sought dental care only after symptoms developed further indicates that preventive dental visits remain underutilized.

Another objective of this study was to explore the relationship between caries severity and caries activity. Previous studies have demonstrated that 75% of dental caries in the primary dentition occur in merely 8.1% of the population, highlighting the importance of identifying high-risk individuals and implementing targeted preventive strategies.^22^ Cariostat is a widely used caries activity test that evaluates the acidogenic potential of dental plaque and has shown value in predicting future caries risk.^5,19,33^

In the present study, both the proportion of high-risk children and the mean Cariostat values increased statistically significantly with caries severity, confirming a positive association between caries activity and clinical caries status. However, a proportion of children with severe caries exhibited low Cariostat values, whereas some caries-free children were classified as high risk. Similar findings have been reported previously,^19,27,33^ suggesting that there is no absolute correspondence between biological caries activity and clinical manifestations.

One explanation for this discrepancy is that Cariostat reflects the current biological activity of the oral biofilm rather than accumulated disease experience. Caries is a multifactorial condition influenced by microbial, host, behavioral, and environmental factors. Consequently, biological risk indicators and clinical disease status do not always coincide. This observation further supports the view that no single indicator can fully characterize an individual’s caries risk.^18^

An important limitation of the present study is the lack of assessment of initial enamel caries lesions and lesion activity. From clinical, epidemiological, and research perspectives, both enamel lesions and caries activity are important factors in ECC. Non-surgical intervention for early-stage lesions (enamel) and surgical intervention for advanced lesions (dentine) are both important for the treatment of caries in young children. The dmfs index was used to maintain comparability with previous regional surveys, and due to practical constraints in kindergartens, such as the lack of professional drying and cleaning tools and the limited cooperation of young children. Future studies should combine lesion activity assessment systems, such as the Nyvad criteria or ICDAS approach, with biological caries activity testing to achieve a more comprehensive evaluation of ECC progression and risk.^3,4^

There are also other limitations. First, the cross-sectional design allows identification of associations between ECC and related factors but does not permit causal inference. Second, information on feeding practices and oral health behaviors was obtained through parental questionnaires and may therefore be subject to recall bias or social desirability bias. Third, participants were predominantly recruited from older urban districts of Jinan, which may limit the generalizability of the findings to other geographic regions or populations. Finally, the questionnaire did not distinguish among different types of milk or beverages consumed at night, nor did it collect detailed information on sugar content. Therefore, the observed association should be interpreted as relating to nighttime feeding behaviors in general rather than to any specific milk composition.

## CONCLUSION

This study reveals a high prevalence of ECC among children aged 3–5 years in Jinan. Effective prevention of ECC should emphasize dietary control, appropriate feeding practices, and the establishment of good oral hygiene habits. Reducing the frequency of sugary food consumption, nighttime feeding, and sleeping with a nipple in the mouth may contribute to lowering ECC risk.

The Cariostat test may serve as a useful adjunctive tool for assessing ECC risk in young children; however, it should not be regarded as a substitute for comprehensive clinical examination. A child’s caries risk cannot be determined solely on the basis of Cariostat values. Regular oral examinations together with comprehensive caries risk assessments are essential for individualized prevention, early intervention, and long-term caries management.

## ACKNOWLEDGEMENTS 

The study was funded by Jinan Stomatological Hospital (grant number: JNSKQYY-2024-03, JNSKQYY-2022-05).
